# The Journal

**Published:** 1854-07

**Authors:** 


### THE JOURNAL.

We intimated in our last issue that we contemplated an enlargement. Our reasons for so doing were founded upon a desire to make it more useful to the profession. Many valuable articles, the choicest productions of medical literature, are usually too lengthy for a Journal of thirty-two pages. In order then that our readers may have the benefit of these essays, monographs, and reviews, we have concluded to enlarge to 80 pages. The only objection to this plan is that when increased to this size, we will be compelled to issue it once every two months, the price of subscription as before, and just so soon as the subscription will justify it, we will issue monthly at the same rates. Our friends are assured that we feel much encouraged at our prospects in the future and as our subscription is increasing we feel profoundly grateful for the "aid and comfort" they have rendered us in the enterprize.

We will then issuse the first No. of the 2d volume on the first or by the middle of the ensuing month in the new and improved form. It will be more than double the size of the monthly and at the end of the year will make a volume of about 156 more pages. Our readers will doubtless be gratified with the improvement in the work, as well as to know that we have redeemed the promise we made upon the outset, that we were determined to enlarge it, and we will now pledge ourselves that so soon as double the number of subscribers now upon our list is obtained, we will make it a monthly. Let then every subscriber who desires to see a permanent journal in the State, and aid an enterprise so important as this to the profession, procure one additional subscriber and soon a monthly will be projected. But we need some of the vitalizing influences as requisites to further growth and we would appeal to our friends who know themselves indebted for the last volume to forward as early as possible the price of subscription.

